# Low Prevalence of Pneumococcal Carriage and High Serotype and Genotype Diversity among Adults over 60 Years of Age Living in Portugal

**DOI:** 10.1371/journal.pone.0090974

**Published:** 2014-03-06

**Authors:** Sónia T. Almeida, Sónia Nunes, Ana Cristina Santos Paulo, Idalina Valadares, Sara Martins, Fátima Breia, António Brito-Avô, Ana Morais, Hermínia de Lencastre, Raquel Sá-Leão

**Affiliations:** 1 Laboratory of Molecular Microbiology of Human Pathogens, Instituto de Tecnologia Química e Biológica, Universidade Nova de Lisboa, Oeiras, Portugal; 2 Agrupamento de Centros de Saúde Oeiras - Carnaxide, Oeiras, Portugal; 3 Agrupamento de Centros de Saúde Alentejo Central II, Évora, Portugal; 4 Laboratory of Molecular Genetics, Instituto de Tecnologia Química e Biológica, Universidade Nova de Lisboa, Oeiras, Portugal; 5 Laboratory of Microbiology of Infectious Diseases, The Rockefeller University, New York, New York, United States of America; School of Medicine, Univ. Complutense, Spain

## Abstract

Pneumococcal disease is frequent at the extremes of age. While several studies have looked at colonization among young children, much less is known among the elderly. We aimed to evaluate pneumococcal carriage among elderly adults living in Portugal. Between April 2010 and December 2012, nasopharyngeal and oropharyngeal swabs of adults over 60 years of age, living in an urban area (n = 1,945) or in a rural area (n = 1,416), were obtained. Pneumococci were isolated by culture-based standard procedures, identified by optochin susceptibility, bile solubility and PCR screening for *lytA* and *cpsA*, and characterized by antibiotype, serotype, and MLST. Associations between pneumococcal carriage, socio-demographic and clinical characteristics were evaluated by univariate analysis and multiple logistic regression. The global prevalence of carriage was 2.3% (95% CI: 1.8–2.8). In the multiple logistic regression analysis, smoking, being at a retirement home, and living in a rural area increased the odds of being a pneumococcal carrier by 4.4-fold (95% CI: 1.9–9.2), 2.0-fold (95% CI: 1.1–3.6) and 2.0-fold (95% CI: 1.2–3.5), respectively. Among the 77 pneumococcal isolates, 26 serotypes and 40 STs were identified. The most prevalent serotypes were (in decreasing order) 19A, 6C, 22F, 23A, 35F, 11A, and 23B, which accounted, in total, for 60.0% of the isolates. Most isolates (93.5%) had STs previously described in the MLST database. Resistance to macrolides, non-susceptibility to penicillin and multidrug resistance were found in 19.5%, 11.7%, and 15.6% of the isolates, respectively. We conclude that the prevalence of pneumococcal carriage in the elderly, in Portugal, as determined by culture-based methods, is low. Serotype and genotype diversity is high. Living in a rural area, in a retirement home, and being a smoker increased the risk of pneumococcal carriage. This study contributes to the establishment of a baseline that may be used to monitor how novel pneumococcal vaccines impact on colonization among the elderly.

## Introduction

*Streptococcus pneumoniae* (or pneumococcus) remains a leading cause of infectious diseases worldwide. The incidence of invasive pneumococcal disease is age-dependent being highest at the extremes of age [1]. Every year, close to 1 million children under the age of 5 die due to pneumococcal infection [2]. The burden of disease among adults is significantly higher but remains poorly defined [1]. Still, infection, per se, is a rare event in the lifecycle of this bacterium and is considered incidental. By contrast, colonization, of the nasopharynx and oropharynx, is frequent and asymptomatic [3].

The study of colonization is essential to understand the biology of pneumococcus for three main reasons: (i) colonization precedes disease, without this prerequisite no infection occurs; (ii) colonization is essential for transmission; (iii) sustainable evolution of the species occurs during colonization (through horizontal gene transfer with other pneumococci and closely related species) [3].

Children are colonized by pneumococci multiple times in the first years of life, frequently at high density, and the prevalence of carriage in this group often exceeds 60% [4], [5]. Less is known about the carrier state among the elderly. As such, the link between carriage and disease in this latter age group remains poorly understood. However, this is increasingly important as the life expectancy of the human population continues to increase and novel strategies to extend the number of quality-adjusted life years among the elderly are needed [6]. In particular, in Europe, it is expected that by 2050, 30.3% of the population will be over 65 years of age (compared to 15.7% in 2000) [7]. In addition, the global annual costs associated to pneumonia in Europe have been estimated in 10 billion euros [6], [8].

Since 2000, multivalent (7-, 10- and 13-valent) pneumococcal conjugate vaccines (PCVs) that prevent disease and colonization by vaccine types have been licensed for use among young children. Recently, a 13-valent pneumococcal conjugate vaccine (PCV13) has been approved for adults over 50 years of age and is now approved for all ages.

In Portugal, PCVs have been commercially available since 2001. The 7-valent PCV (PCV7) became available in June 2001; the 10- and 13-valent PCVs (PCV10 and PCV13) became available in April 2009 and January of 2010 (PCV13 replaced PCV7), respectively. PCVs have not been introduced in the National Immunization Plan, nor are reimbursed by the state. Estimate usage based on national sales indicates a gradual increase in the use of PCV7 reaching 75% in 2008. The impact of PCV7 in colonization and disease has been documented [9]–[11]. In 2010, when the fieldwork described in this study began, the estimated use of PCV10 and PCV13 in infants was 7% and 58%, respectively (IMS and INE/National Statistics Institute). PCV13 was approved for adults over 50 years of age in January 2012 and available data indicate its use has been very low (c.a. 1%, Health Market Research).

In Portugal, pneumococcal colonization in young children has been studied for over 15 years in the urban areas of Lisbon and Oeiras [11]–[14]. By contrast, pneumococcal colonization patterns among the elderly have not been described.

In this study, conducted between 2010 and 2012, we aimed to gain insights on pneumococcal colonization patterns among the elderly in order to establish a baseline before conjugate vaccines became widely used in this age group. Specific objectives were: (i) to estimate the prevalence of pneumococcal colonization among adults aged over 60-years of age; (ii) to determine risk factors associated with pneumococcal colonization; (iii) to characterize colonizing strains by antibiotype, serotype and MLST; and (iv) to estimate potential coverage of pneumococcal vaccines.

## Methods

### Ethics Statement

The study was registered and approved at health care centers of Oeiras and Montemor-o-Novo that report to Administração Regional de Saúde (ARS, “Regional Health Administration”) of Lisboa e Vale do Tejo, and Alentejo, respectively, from the Ministry of Health. The study was approved by the directors of all participating institutions. Informed written consent was obtained from all participants. All samples and questionnaires were numerically coded upon sample collection and processed anonymously. Data was stored in a dedicated secure database.

### Study Population

Adults older than 60 years of age, living in Oeiras, an urban area, or in Montemor-o-Novo, a rural area of Portugal, were invited to participate in the study. The municipality of Oeiras has a population of over 160,000 inhabitants of which 15% are over 65 years of age, and a population density of 3,537/km^2^. Oeiras is located next to the capital, Lisbon, in the Atlantic Coast. By contrast, the municipality of Montemor-o-Novo is located away from the sea in the most deserted region of the country, Alentejo. It has a population of 18,500 inhabitants of which 26% are over 65 years of age, and a population density of 13/km^2^ [15].

### Study Design

Sampling was carried out between April 2010 and December of 2012. We planned to enroll 3,200 subjects assuming an estimated prevalence of pneumococcal carriage in risk groups of 3.0% and an odds ratio to be colonized of 2 with a two-sided confidence interval of 95% and a power of 80%.

Inclusion criteria were living in the area under study, age over 60 years, and willingness to participate and provide informed written consent prior to enrollment. On the day of sample collection, participants were interviewed to obtain socio-demographic and medical history data. This information was registered in a standard questionnaire. For each adult one nasopharyngeal and one oropharyngeal sample were obtained.

In both areas most individuals were approached in the local health care center. In addition, day centers and retirement homes were also visited (eight in the rural area and twenty in the urban area). Health care centers are part of the National Health System and serve the entire Portuguese population providing primary care and routine consultations.

### Sampling and Isolation

Nasopharyngeal and oropharyngeal samples were collected as previously described using cotton and viscose swabs, respectively [16]. Swabs were placed in Stuart transport media, and were maintained at room temperature for 2–30 hours. All samples were processed in our laboratory. Swabs were streaked on gentamycin blood agar and incubated for 24 h at 37°C in anaerobiose jars. On the following day, suspected pneumococcal colonies were isolated and identified following routine procedures based on optochin susceptibility and bile solubility [13], [17]. Additionally, isolates showing one or more atypical results in the latter assays but displaying a characteristic pneumococcal colony morphology were further characterized by a strategy recently developed based on a multiplex polymerase chain reaction targeting *lytA*, *cpsA*, *aliB-like* ORF2, and *16SrDNA* genes, followed, if needed, by identification of *lytA* pneumococcal-specific RFLP signatures as described by Llull *et al.* [18], [19]. The swabs and the pure cultures were frozen and stored at −80°C in STGG [20].

### Capsular Typing

Isolates were typed by multiplex PCR using primers previously described [21] and www.cdc.gov and/or by the Quellung reaction using commercially available pneumococcal antisera (Statens Serum Institute, Copenhagen, Denmark) [22].

### Antimicrobial Susceptibility Testing

Antimicrobial susceptibility testing was performed using the Kirby-Bauer technique, according to the CLSI recommendations and definitions [23]. The antimicrobial agents tested were chloramphenicol, erythromycin, clindamycin, tetracycline, and sulfamethoxazole-trimethoprim (SXT). Antibiotic disks were purchased from Oxoid (Hampshire, England). Interpretation of results followed the CLSI guidelines [23]. Isolates were also screened for MICs to penicillin (only for oxacillin non-susceptible isolates) and ciprofloxacin (all isolates) with the E-test (AB Biodisk, Solna, Sweden) according to the manufacturer’s recommendations. In the interpretation of decreased penicillin susceptibility epidemiological breakpoints were used: isolates were considered intermediately resistant if the MIC was ≥0.1 µg/ml and <1.5 µg/ml and were considered resistant if the MIC was ≥1.5 µg/ml. For ciprofloxacin, strains with MIC≥4 µg/ml were considered resistant [24].

### Multilocus Sequence Typing (MLST)

MLST was done essentially as previously described [25] using primers with universal M13 tails. Sequencing reactions were performed at Macrogen (Amsterdam, The Netherlands). Sequences were analyzed with the Bionumerics software (Applied Maths, Gent, Belgium). Allele numbers and sequence types (ST) were determined using the website http://www.mlst.net for *Streptococcus pneumoniae*.

### Statistical Analysis

Descriptive statistics (mean and standard deviation or percentages as appropriate) were used to summarize each of the socio-demographic and clinical variables included in the questionnaire. A univariate logistic regression was used as an exploratory analysis to select for the variables associated with colonization (yes/no). At this step we used a p-value of <0.05. The crude odds ratios (OR) were estimated from the univariate logistic regression. The variables that were significantly associated with the outcome were included in a multiple logistic regression which was carried out to statistically adjust for potential differences in the distribution of the independent variables [26]. The model that had the lowest AIC (Akaike Information Criterion) was regarded as the most parsimonious model. The goodness-of-fit of the final model was further explored using the Hosmer-Lemeshow test [26]. From the final model we estimated the adjusted OR and the corresponding 95% CI. We considered a variable to be significantly associated with the outcome when the adjusted 95% CI odds ratio did not include 1.0. A change in 10% between the value of the crude OR and the adjusted OR was considered to signal a confounder. As the number of carriers was low compared to the total number of subjects sampled, we compared the coefficients estimated by bootstrapping a multiple logistic regression to ensure that the classical estimate had the minimum bias and standard error since it could compromise the estimates of the confidence interval.

The Gini-Simpson index of diversity (GSID) was used to calculate serotype and genotype diversity in the population. All the analyses were carried out using R (version 2.15.0) [27].

## Results

### Characteristics of the Study Population

A total of 3,361 adults over 60 years of age participated in the study (Table 1); 1,416 (42.1%) lived in the rural area and 1,945 (57.9%) in the urban area. The mean age of the participants was 74.5±8.2 years old (range 60–104 yrs old). Females were overrepresented (57.6%). Most participants had very few years of formal education with only 7.3% having more than four years. Most participants were retirees (89.7%) and lived in the family home (91.1%, average household size of 2.2±1.1). Close to one-fifth had weekly contact with young children and one-third engaged in regular recreational activities (Table 1).

**Table 1:** Socio-demographic characteristics and potential risk factors for pneumococcal carriage.

Characteristic	Participants n (%)	No. pneumococcal carriers n (% among participants with that characteristic)	p-value
**Living area**			**<0.001**
urban	1945 (57.9%)	28 (1.4%)	
rural	1416 (42.1%)	48 (3.4%)	
**Gender**			0.115
female	1935 (57.6%)	37 (1.9%)	
male	1426 (42.4%)	39 (2.7%)	
**Years of school education** a			0.305
0	315 (9.4%)	11 (3.5%)	
1–4	2799 (83.3%)	60 (2.1%)	
≥5	246 (7.3%)	5 (2.0%)	
**Retirees**			0.286
retired	3015 (89.7%)	71 (2.4%)	
active	346 (10.3%)	5 (1.4%)	
**Housing**			**<0.001**
family home	3062 (91.1%)	59 (1.9%)	
retirement home	299 (8.9%)	17 (5.7%)	
**Weekly contact with children** **≤6 yrs**			0.929
yes	650 (19.3%)	15 (2.3%)	
no	2711 (80.7%)	61 (2.3%)	
**Recreational activities**			0.230
at least one activity	1119 (33.3%)	25 (2.2%)	
club	339 (10.1%)	9 (2.7%)	
day center	652 (19.4%)	14 (2.1%)	
senior university	51 (1.5%)	2 (3.9%)	
other	99 (2.9%)	0 (0.0%)	
**Smoker**			**0.003**
yes	126 (3.7%)	8 (6.3%)	
no	3235 (96.3%)	68 (2.1%)	

a not available for one participant, reference test was 0 years of school education.

Smoking was uncommon with only 3.7% of the participants being active smokers (Table 1). Regarding medical history, most participants (82.6%) had at least one chronic disease being the most frequent hypertension (61.2%), heart disease (32.2%), and diabetes (28.3%). In the previous year, 15.2% had been admitted in a hospital for at least one night and 45.3% had had respiratory infection(s). At the time of sampling, 38.6% had one or more symptoms of respiratory infection as described in Table 2.

**Table 2:** Medical conditions and risk factors for pneumococcal carriage.

Characteristic	Participants n (%)	No. pneumococcal carriers n(% among participants with that characteristic)	p-value
**Chronic disease**			
at least one of the indicated below	2776 (82.6%)	65 (2.3%)	0.498
none	585 (17.4%)	11 (1.9%)	
**COPD**			**0.013**
yes	497 (14.8%)	19 (3.8%)	
no	2864 (85.2%)	57 (25.0%)	
**asthma**			**0.020**
yes	218 (6.5%)	10 (4.6%)	
no	3143 (93.5%)	66 (2.1%)	
**hepatic disease**			0.259
yes	70 (2.1%)	3 (4.3%)	
no	3291 (97.9%)	73 (2.2%)	
**renal disease**			0.704
yes	68 (2.0%)	2 (2.9%)	
no	3293 (98.0%)	74 (2.2%)	
**diabetes**			0.897
yes	951 (28.3%)	20 (2.1%)	
no	2410 (71.7%)	56 (2.3%)	
**hypertension**			0.281
yes	2058 (61.2%)	41 (2.0%)	
no	1303 (38.8%)	35 (2.7%)	
**heart disease**			0.269
yes	1082 (32.2%)	20 (1.8%)	
no	2279 (67.8%)	56 (2.5%)	
**Hospitalization in previous year**			0.414
yes	510 (15.2%)	9 (1.8%)	
no	2851 (84.8%)	67 (2.4%)	
**Respiratory infection in previous year**			0.080
at least one of the indicated below	1524 (45.3%)	42 (2.8%)	
none	1837 (54.7%)	34 (1.9%)	
**asthma/bronchitis**			**<0.001**
yes	200 (6.0%)	12 (6.0%)	
no	3161 (94.0%)	64 (2.0%)	
**cold/flu**			**0.033**
yes	997 (29.7%)	31 (3.1%)	
no	2364 (70.3%)	45 (1.9%)	
**rhinosinusitis**			0.119
yes	367 (11.0%)	4 (1.1%)	
no	2994 (89.0%)	72 (3.1%)	
**tonsillitis**			0.481
yes	359 (10.7%)	10 (2.8%)	
no	3002 (89.3%)	66 (2.2%)	
**pneumonia**			0.640
yes	64 (1.9%)	2 (3.1%)	
no	3297 (98.1%)	74 (2.2%)	
**Mild symptoms of respiratory disease at sampling**			
at least one of the indicated below	1298 (38.6%)	38 (2.9%)	**0.040**
none	2063 (61.4%)	38 (1.8%)	
**sputum**			**0.013**
yes	330 (9.8%)	14 (4.2%)	
no	3031 (90.2%)	62 (2.0%)	
**cough**			**0.006**
yes	330 (9.8%)	23 (3.9%)	
no	3031 (90.2%)	53 (1.7%)	
**shortness of breath**			**<0.001**
yes	278 (8.3%)	15 (5.4%)	
no	3083 (91.7%)	61 (2.0%)	
**sore throat**			0.203
yes	491 (14.6%)	15 (3.1%)	
no	2870 (85.4%)	61 (2.1%)	
**runny nose**			0.129
yes	425 (12.6%)	14 (3.3%)	
no	2936 (87.4%)	62 (2.1%)	
**fever**			0.393
yes	19 (0.6%)	1 (5.3%)	
no	3342 (99.4%)	75 (2.2%)	
**Vaccination**			
**seasonal flu**			0.720
yes	1969 (58.6%)	43 (2.2%)	
no	1392 (41.4%)	33 (2.4%)	
**PPV23** a			0.444
yes	122 (3.6%)	4 (3.3%)	
no	3239 (96.4%)	72 (2.2%)	
**Antibiotic consumption**			
**at sampling**			0.978
yes	62 (1.8%)	0 (0.0%)	
no	3299 (98.2%)	76 (2.3%)	
**month before**			0.511
yes	191 (5.7%)	3 (1.6%)	
no	3170 (94.3%)	73 (2.3%)	
**previous 6 months**			0.070
yes	257 (7.6%)	1 (0.4%)	
no	3104 (92.4)	75 (2.4)	

a 23-valent pneumococcal polysaccharide vaccine.

Over half (58.6%) of the participants had received the seasonal flu vaccine and only 3.6% had received 23-valent pneumococcal polysaccharide vaccine (PPV23). Antibiotic consumption was low with 7.6% of the participants reporting taking antibiotic in the previous six months. Urinary infections were the main reason for taking an antibiotic (data not shown).

### Pneumococcal Carriage

Of the 3,361 elderly, 188 presumptive pneumococci were isolated based on optochin susceptibility or bile solubility. However, further testing based on a multiplex PCR assay [19], RFLP-*lytA* signatures [18] and, if needed, MLST, led to the exclusion of 111 isolates. Overall, 77 isolates were confirmed as pneumococci and these were isolates from 76 (2.3%) carriers. One subject carried two different isolates in the nasopharynx that were identified based on distinct colony morphology; both were isolated, serotyped and genotyped as described in the next section. In total, pneumococci were isolated from 18 oropharyngeal samples and 64 in nasopharyngeal samples; in 6 participants pneumococci were isolated from both sites and the paired isolates were found to be identical. There was no significant difference in the prevalence of colonization over time (p = 0.392), which was 2.2% (95% CI: 1.6–3.2) in 2010, 2.6% (95% CI: 1.8–3.6) in 2011, and 1.6% (95% CI: 0.9–2.8) in 2012.

In the univariate analysis, there were significantly more carriers in the rural area than in the urban area [48/1416 (3.4%) vs 28/1945 (1.4%), respectively, p<0.001]. Carriage was also significantly higher among participants living in retirement homes than among those who lived in the family home [59/3062 (5.7%) vs 17/299 (1.9%), p<0.001], smokers [8/126 (6.3%) vs 68/3235 (2.1%), p = 0.003], those with COPD [19/497 (3.8%) vs 57/2864 (2.0%), p = 0.013] or asthma [10/218 (4.6%) vs 66/3143 (2.1%), p = 0.020], those who reported having asthma/bronchitis [12/200 (6.0%) vs 64/3161 (2.0%), p<0.001], cold/flu [31/997 (3.1%) vs 45/2364 (1.9%), p = 0.033] in the previous year, and participants with sputum [14/330 (4.2%) vs 62/3031 (1.2%), p = 0.013), cough [23/330 (3.9%) vs 53/3031 (1.6%), p = 0.006] or shortness of breath [15/278 (5.4%) vs 61/3083 (2.0%), p<0.001] at time of sampling (Tables 1 and 2).

After multivariate logistic regression analysis, living in the rural area (OR = 2.0, 95% CI: 1.2–3.5), being at a retirement home (OR = 2.0, 95% CI: 1.1–3.6) and smoking (OR = 4.4, 95% CI: 1.9–9.2) increased significantly the odds of being a pneumococcal carrier (Table 3).

**Table 3:** Odds ratio and adjusted odds ratio of factors associated with pneumococcal carriage among adults older than 60 years of age.

Variable	Unadjusted OR (95% CI)	Adjusted OR (95% CI)
Area		
urban	1	1
rural	2.4 (1.5–3.8)	**2.0 (1.2–3.5)**
Housing		
family home	1	1
retirement home	3.1 (1.7–5.2)	**2.0 (1.1–3.6)**
Smoker	3.2 (1.4–6.4)	**4.4 (1.9–9.2)**
Chronic disease		
COPD	1.9 (1.1–3.2)	0.9 (0.5–1.7)
asthma	2.2 (1.1–4.2)	1.2 (0.5–2.5)
Respiratory infection in previous year		
asthma/bronchitis	3.1 (1.6–5.6)	1.8 (0.8–4.0)
cold/flu	1.7 (1.0–2.6)	1.3 (0.8–2.1)
Mild symptoms of respiratory disease at sampling		
sputum	2.1 (1.1–3.7)	1.3 (0.6–2.5)
cough	2.0 (1.2–3.2)	1.3 (0.7–2.3)
shortness of breath	2.8 (1.5–4.9)	1.8 (0.9–3.5)

### Characteristics of Pneumococcal Isolates

Among the 77 pneumococcal isolates, 26 serotypes and 40 STs were identified. A high serotype (GSID 0.946, 95% CI: 0.929–0.963) and genotype (GSID 0.970, 95% CI: 0.956–0.984) diversity was detected.

**Figure 1:**
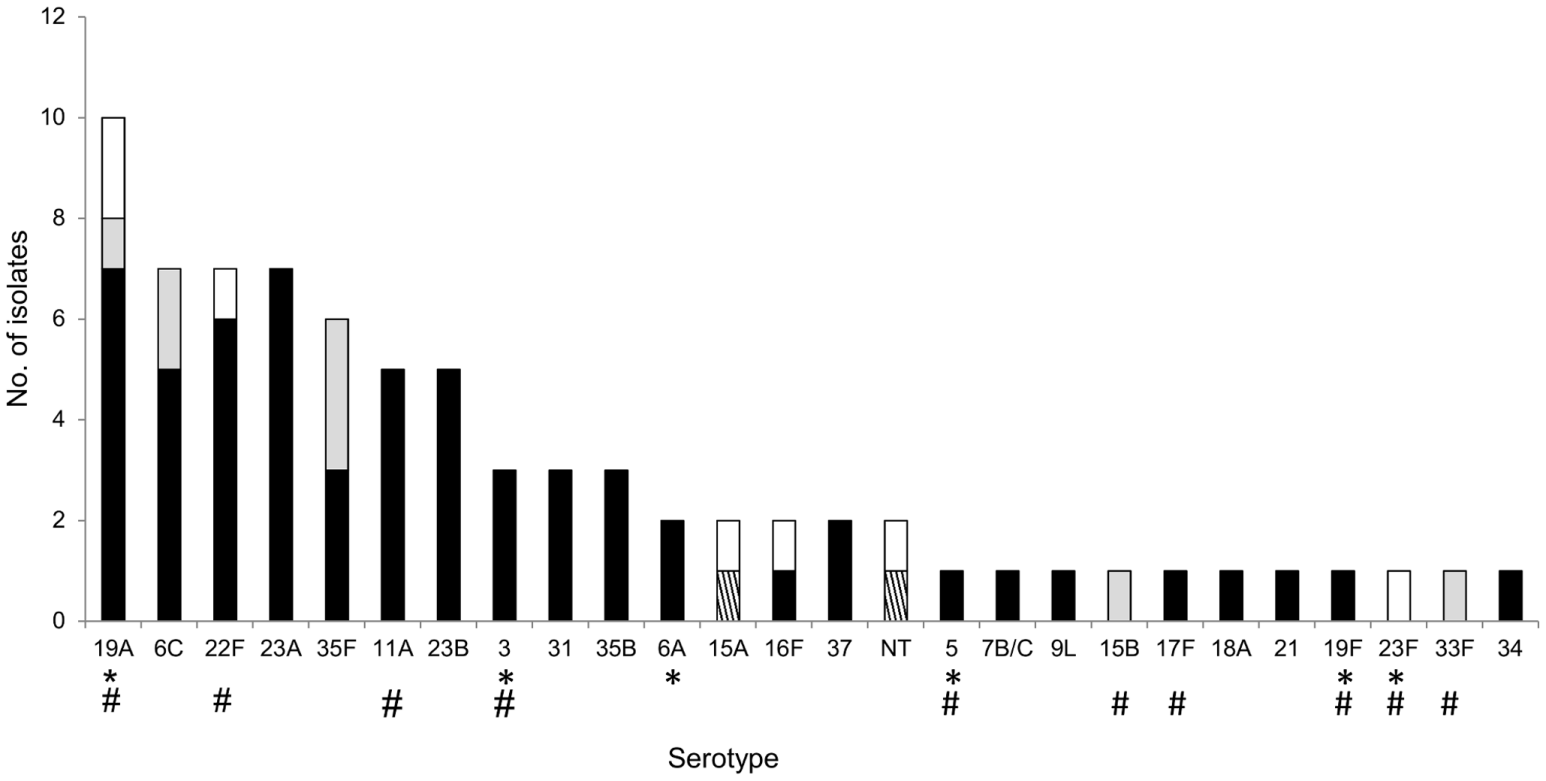
Serotype distribution of pneumococcal isolates carried by adults over 60 years of age. Black bars, isolates susceptible to penicillin and erythromycin. Striped bars, isolates non-susceptible to penicillin; gray bars, isolates resistant to erythromycin; white bars, isolates resistant to erythromycin and non-susceptible to penicillin. * indicates serotypes included in PCV13; # indicates serotypes included in PPV23.

The most prevalent serotypes were (in decreasing order) 19A (12.9%), 6C, 22F and 23A (9.0% each), 35F (7.6%), and 11A, 23B (6.4% each), which, accounted, in total, for 60.0% of the isolates. Overall, 23.4% and 40.3% of the isolates belonged to serotypes included in PCV13 and PPV23, respectively (Figure 1).

**Figure 2:**
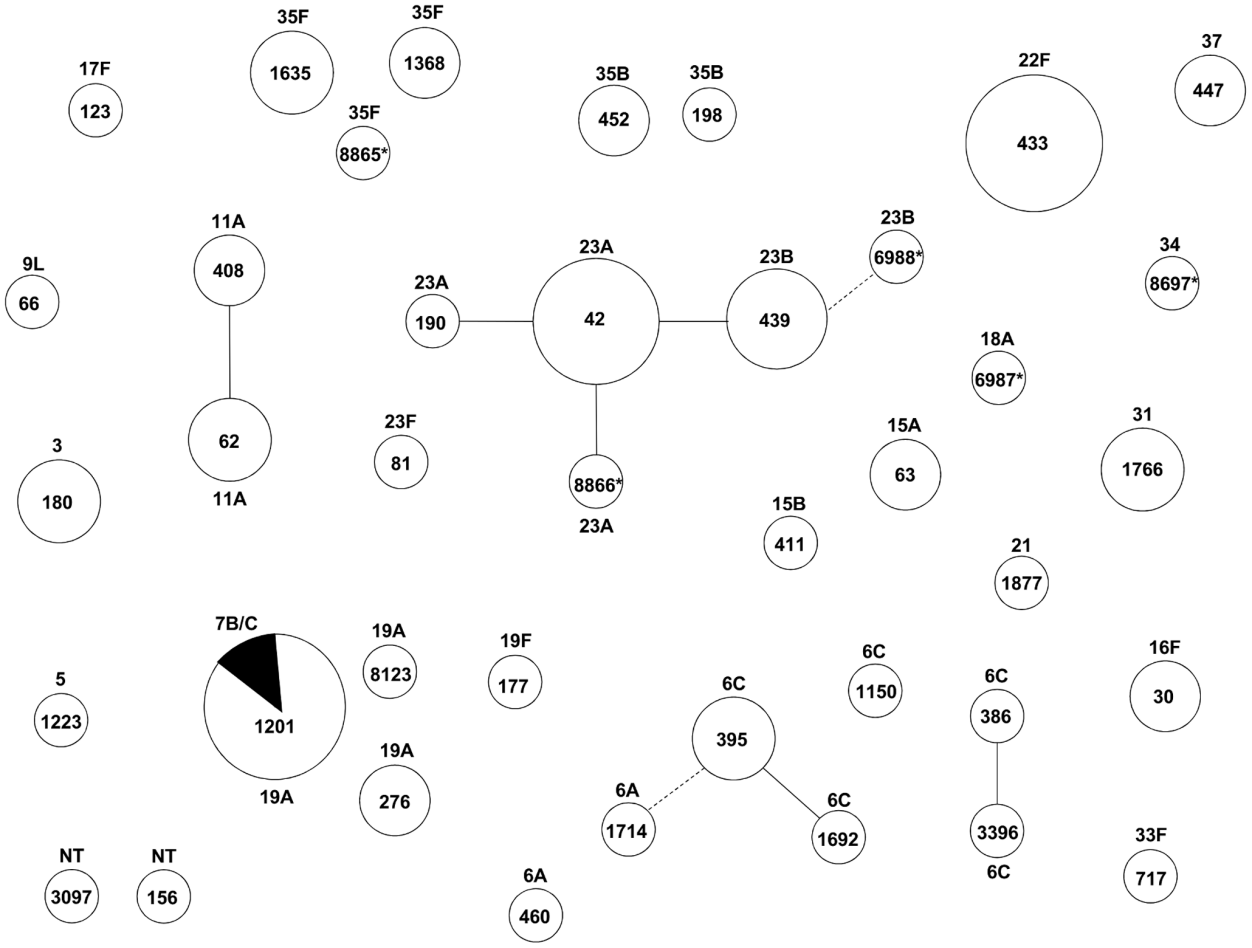
Graphical representation of MLST analysis of the 77 pneumococcal isolates (based on goeBURST). Numbers inside circles indicate sequence type (ST). *indicates novel ST identified in this study. The solid and dotted lines connect single locus variants (SLV) and double locus variants (DLV), respectively. Numbers outside circles indicate serotype. The size of the circles is proportional to the number of isolates of each ST. The smallest circle corresponds to one isolate; the biggest corresponds to seven isolates.

Most isolates (93.5%) had STs previously described in the MLST database and 5 novel STs were identified (6987, 8866, 6988, 8697 and 8865 associated with serotypes 18A, 23A, 23B, 34, and 35F, respectively). These novel STs resulted solely from novel combinations of alleles previously described in the *S. pneumoniae* MLST database (http://spneumoniae.mlst.net). The properties of the strains yielding these novel STs have been deposited at the *S. pneumoniae* MLST database. No major lineages were detected. The largest group of strains sharing related STs contained 12 isolates and was associated to serotypes 23A and 23B (Figure 2).

The two pneumococcal strains isolated from the same carrier detected on the basis of a distinct colony morphology were found to be of serotype 22F and non-typeable and STs 433 and 3097, respectively.

The four PPV23 vaccinated individuals carried serotypes 6C, 35F (n = 2), and 23A. None of these serotypes are included in PCV13 neither in PPV23.

Of all isolates, 37.7% were resistant to at least one antimicrobial agent; twelve isolates (15.6%) were multidrug resistant (i.e. resistant to three or more classes of antimicrobials). The proportion of resistant isolates was 11.7% for penicillin (8 isolates with intermediate resistance and 1 resistant isolate, MIC_50_<0.064 µg/ml, MIC_90_ = 0.094 µg/ml, range: <0.064–1.5 µg/ml), 19.5% to macrolides (13 out of 15 macrolide resistant isolates were of MLSb phenotype), 16.9% to tetracycline, 7.8% to SXT, 2.6% to chloramphenicol, and 1.3% to ciprofloxacin (MIC_50_ = 0.75 µg/ml, MIC_90_ = 1.5 µg/ml, range: 0.25–8 µg/ml) (Table S1). Serotypes associated with resistance to macrolides and/or non-susceptibility to penicillin are shown in Figure 1.

## Discussion

To our knowledge this is the first study conducted in Portugal aiming to evaluate pneumococcal carriage in the elderly population. To improve isolation of pneumococci, nasopharyngeal and oropharyngeal swabs were used as previously suggested [16]. Studies from other countries such as Belgium, Finland, Israel, and Kenya, described low prevalence of colonization (typically up to 5%) among adults [28]–[31]. Of note, only the studies from Finland and Israel have described the serotype diversity and found that 71% (of 31) and 45% (of 38) of the isolates, respectively, were non-typeable. The remaining isolates had diverse capsular types [30], [31]. In our study, the prevalence of pneumococcal colonization among adults over 60 year of age was also low (2.3%); and serotype and genotype diversity were high in line with previous observations [30], [31] except for the fact that a lower proportion of non-typeables was found (2.6% of 77). Together, our observations suggest that transmission chains within the elderly are not frequent and alternative reservoirs (such as young children) may be the main sources of pneumococci.

Of interest, a recent study where healthy adults were challenged with a live serotype 6B pneumococcal strain, suggested that carriage in healthy adults works as a natural boosting mechanism to maintain the protective immunity against disease. The authors postulated that, in the elderly, the apparent impaired capacity to establish colonization, that results in low carriage prevalence in this age group, may be due to the increased production of inflammatory mediators (inflammaging) resulting in the disruption of a balanced immune response needed for carriage persistence. This lack of stimulation of natural immunity might explain the high incidence of invasive pneumococcal disease among the elderly [32].

In our population the risk of being a pneumococcal carrier was found to be higher among smokers (4.4-fold), those living in a retirement home (2.0-fold), and those living in the rural area (2.0-fold).

Smoking is a known risk factor for invasive pneumococcal disease [33] and parental smoking has been associated with increased risk of otitis media in children [34]. Cigarette smoking has been previously identified as a risk factor for pneumococcal carriage among patients with HIV-1 and among mothers of young children [35], [36]. Passive smoking in adolescents and children [33], [36] and frequent exposure to outside fires among Australian Aboriginal adult population [37] have also been described as risk factors for pneumococcal carriage. In our study, cigarette smoking was the strongest risk factor for pneumococcal carriage in the elderly.

Living in a retirement home has been previously associated with increased pneumococcal carriage and these settings have been implicated in outbreaks of pneumococcal disease [35], [37], [38]. Why living in a rural area increased the risk of carriage in our study is not self-evident. One may hypothesize that other factors that could explain this observation were not studied. Also, in other studies, COPD or asthma, cold/flu, sputum, cough and shortness of breath were previously identified as risk factors for pneumococcal carriage [31], [37]. In our study, in the univariate analysis, we also identified these variables as risk factors for carriage but these lost significance when multiple logistic regression to control for possible confounders was done.

We found relatively low theoretical coverages of PPV23 (40.3%) and PCV13 (23.4%). The limited coverage of these vaccines may be, at least in part, explained by the wide use of PCV7 among Portuguese children that has resulted in a significant decline of PCV7 serotypes both in carriage and disease [9]–[11].

Of interest, after correct identification of all presumptive pneumococci, only two (2.6%) non-typeable true pneumococci were identified. Our results contrast with other studies that reported, among adult carriers, a large proportion (>20%) of non-typeable pneumococci [30], [31]. They also raise the question on whether correct identification of such isolates is being done across studies. Identification of non-typeable pneumococci is prone to errors as close relatives such as *S. pseudopneumoniae* and *S. mitis* may give false positive results in routine tests [39]–[41]. In our study, all isolates were unambiguously identified and 112 putative non-capsulated pneumococcal isolates were excluded after detailed analysis.

Our study has some limitations. First, our sample was a convenience sample, which has inherent limitations, as it may not be representative of the population approached. Still, individuals were approached at health care centers, day care centers and retirement homes, and therefore a diverse population has been included in the study, even if not selected at random. Secondly, although the size of the population enrolled was high (3,361 subjects), the low number of pneumococcal isolates identified may have hindered the statistical analysis and the study may have been underpowered to detect additional risk factors for carriage. Thirdly, we only used culture-based methods to detect pneumococci, which may have resulted in a lower carriage prevalence estimate. While we used a selective medium to prevent overgrowth of undesirable bacteria, we may have been unable to detect low-density carriers. In fact, Ogami *et al.* reported that real-time PCR increased significantly detection of carriage in healthy children when compared to culture alone [42]. The same rationale is plausible for detection of carriage in adults. More recently, Chien *et al.* reported that at a carriage density of ≤10^5^ CFU/ml, detection of pneumococci by culture was significantly less sensitive than by real-time PCR [43]. Also, Trzciński *et al.* have suggested that carriage prevalence in adults is underestimated, and reported that oropharyngeal carriage prevalence was significantly higher than generally reported when qPCR after culture-enrichment was used (with a prevalence of c.a. 40% among parents of young children) [44]. Almost simultaneously, a study by Carvalho *et al*. suggested that, in adults, pneumococci are mostly found at the nasopharynx [45]. The same group reported that high rates of false positives can be found in the oropharynx if only molecular methods are used for pneumococcal detection and serotyping [46].

Our study has also some strength. As all pneumococci were isolated and cultured it was possible to unequivocally identify them preventing detection of false positives. MLST analysis of all isolates also confirmed that all were pneumococci.

Overall, while it is clear that molecular methods have superior sensitivity for bacterial detection, it is also becoming obvious that their specificity may be compromised due to difficulties in establishing species boundaries between pneumococcus and closely related species [19], [40], [47]. Clearly, at the present time, a combination of highly sensitive methods with culture isolation and unequivocal identification of pneumococci may be the best solution to clarify controversial findings. In our laboratory, we are currently addressing these issues.

In conclusion, this study showed for the first time that pneumococcal colonization (detected on the basis of culture methods) in the elderly, in Portugal, is low. Colonizing isolates are very diverse in terms of serotype and genotype. Living in a rural area, living in a retirement home and smoking increased the risk of being colonized with pneumococci. This study will contribute to establish a baseline that may be used in future studies to monitor how novel pneumococcal vaccines impact on colonization among the elderly. This novel information should help decision makers and public health authorities in defining strategies aimed to prevent pneumococcal disease among the elderly.

## Supporting Information

Table S1 Antimicrobial resistance according to serotype.
(DOCX)
