# Supplementary material for: Low Prevalence of Pneumococcal Carriage and High Serotype and Genotype Diversity among Adults over 60 Years of Age Living in Portugal
Source: PLoS One. 2014 Mar 6;9(3):e90974. doi: 10.1371/journal.pone.0090974 (PMC3946249; doi:10.1371/journal.pone.0090974)
Supplement: Table S1 — Antimicrobial resistance according to serotype. (DOCX) [file pone.0090974.s001.docx]

**TABLE S1.** Antimicrobial resistance according to serotype

| **Serotype** | **No.of isolates** | **Antibiotype (no. of isolates)** |
| --- | --- | --- |
| 3 | 3 | susceptible |
| 5 | 1 | susceptible |
| 6A | 2 | Tet (1) |
| 6C | 7 | Ery (2), Cc (2), Tet (2) |
| 7B/C | 1 | susceptible |
| 9L | 1 | susceptible |
| 11A | 5 | SXT (1), Cip (1) |
| 15A | 2 | ^I^PG (2), Tet (1), Ery (1), Cc (1) |
| 15B | 1 | Ery |
| 16F | 2 | ^I^PG (1), Ery (1), Cc (1), Tet (1) |
| 17F | 1 | susceptible |
| 18A | 1 | susceptible |
| 19A | 10 | ^I^PG (2), Chl (1), Ery (3), Cc (3), Tet (3), SXT (1) |
| 19F | 1 | susceptible |
| 21 | 1 | susceptible |
| 22F | 7 | ^I^PG (1), Ery (1), Cc (1), Tet (1) |
| 23A | 7 | susceptible |
| 23B | 5 | susceptible |
| 23F | 1 | ^R^PG, Chl, Ery, Cc, Tet, SXT |
| 31 | 3 | susceptible |
| 33F | 1 | Ery, Cc, Tet |
| 34 | 1 | SXT |
| 35B | 3 | susceptible |
| 35F | 6 | Ery (3), Cc (2), Tet (1) |
| 37 | 2 | susceptible |
| NT | 2 | ^I^PG (2), Ery (1), Cc (1), Tet (1), SXT (2) |

^I^PG, intermediately resistant to penicillin (0.1 µg/ml ≤ MIC ˂1.5 µg/ml); ^R^PG, resistant to penicillin (MIC ≥1.5 µg/ml); Chl, chloramphenicol; Ery, erythromycin; Cc, clindamycin; Tet, tetracycline; SXT sulfamethoxazole-trimethoprim; Cip, ciprofloxacin.
